# Linking the processes of medication administration to medication errors in the elderly

**DOI:** 10.4102/hsag.v27i0.1704

**Published:** 2022-01-31

**Authors:** Emerentia C. Nicholson, Anneleen Damons

**Affiliations:** 1 Department of Nursing and Midwifery, Faculty of Medicine and Health Sciences, Stellenbosch University, Cape Town, South Africa

**Keywords:** elderly, long-term care facilities, medication, medication administration, medication errors, residential facilities

## Abstract

**Background:**

Older people are more prone to chronic diseases than younger ones and typically receive multiple medications. Medication rounds in long-term care facilities (LTCFs) are usually lengthy, with most errors occurring during the administration phase. How nurses apply medication administration processes can affect resident outcomes.

**Aim:**

To determine the processes of medication administration followed by nurses in LTCFs as self-reported by them to identify possible factors associated with medication errors.

**Setting:**

Twenty-eight LTCFs for the elderly in the Western Cape province, South Africa.

**Methods:**

A non-experimental cross-sectional descriptive design was applied, using a quantitative approach. A stratified sampling method obtained equal samples of nurses from funded and private LTCFs, thus *N* = 123 respondents. Data collection was via self-administered questionnaires. The Statistical Package for the Social Sciences (SPSS27) was used for descriptive and inferential analysis.

**Results:**

Nurses’ self-reported medication errors such as the sharing of medication between residents (83%), the omission of doses (64.8%), neglecting to sign after medication administration (57%), and medication administered at the wrong time (50.8%). Frequent interruptions during medication rounds were the most common reason for medication errors (75.6%).

**Conclusion:**

Multiple medication administration process errors were self-reported by the nurses. LTCFs should provide mandatory medication training, monitor the adherence to correct medication administration procedures, and implement risk-management strategies.

**Contribution:**

The identified factors associated with medication errors during medication administration processes can assist with developing risk management strategies and policies in the LTCFs and improve evidence-based practice and resident outcomes.

## Introduction

An unprecedented increase in the ageing of the world’s overall population is noticeable. People who were 65 years of age and older outnumbered children under the age of 5 years for the first time in 2018 (United Nations 2019:1). Ageing comes with specific challenges, such as a deterioration in functional abilities, leading to poor health and chronic diseases (WHO 2015). The increase in chronic diseases leads to multiple medications (Qian et al. 2015). The estimation is that older people take from two to as many as nine different medications per day, which increases the risk of adverse drug events (Dagli & Sharma 2014). These adverse drug events are defined as any unintentional injuries caused by medication that require patient observation or intervention. These injuries could lead to harm, hospitalisation, or death (Al-Jumaili & Doucette 2017). In addition, the symptoms associated with adverse events are often wrongly diagnosed as new diseases, leading to the prescription of even more medications (Dagli & Sharma 2014).

A medication error is any error occurring in the patient’s treatment process, which could lead to or potentially lead to harm to the patient (Al-Jumaili & Doucette 2017). Ferrah, Lovell and Ibrahim (2017) identified antipsychotics, antidiabetics, sedatives, anticoagulants, and diuretics, as the highest contributors to medication errors leading to hospitalisation and death of nursing home residents. Furthermore, when a patient experienced an adverse event resulting from a medication error, the length of hospitalisation increased by 4.6 days, with a cost of $4585 per event (about R71 818.06) (Shah et al. 2016).

The administration of medications to older people encompasses a medication management process consisting of five phases: prescribing and ordering, transcribing, dispensing, administering, and monitoring (Al-Jumaili & Doucette 2017; Ferrah et al. 2017). Ferrah et al. (2017) found that although medication errors occurred in all the phases of medication management, the most severe and preventable errors happened in the medication administration phase. During the administration phase, nurses are challenged by lengthy medication rounds because of multiple medications for each person. Qian et al. (2015) found that 52% of residents in their study took between six and 10 tablets per day, while 67% of the residents needed help from the nurses to take their tablets. The medication rounds required between 2.5 and 4.5 h per day representing 37.5% of the nurses’ time in an 8-h shift (Qian et al. 2015). About 40% of nurses and nurse assistants mentioned interruptions during a medication round as a significant barrier, especially during medication preparation (Dilles et al. 2011). When faced with the challenge of completing a medication round on time to prevent action against them, nurses admit to following compromising practices such as disguising medication in fruit juice to ensure that residents swallow the medication without spitting it out (Ellis et al. 2012). A concern is that certain fruit juices may affect the metabolism of medicines, thus increasing or decreasing the effectiveness of the medication (Preston, Jones & Sandhu 2014).

The South African Nursing Council (SANC) changed the nomenclature of nurses in the *Nursing Act* 33 of 2005 (RSA 2005). Because of the phasing out of the current or legacy qualifications, previous designations are still in use and were also used in this study (RSA 2005; SANC 2021). For clarification, a professional/registered nurse (RPN) will be referred to as a registered nurse (RN), the registered staff nurse (RSN) as an enrolled nurse (EN), and the registered auxiliary nurse (RAN) as an enrolled nurse assistant (ENA). The ENAs are tasked with elementary nursing care. ENAs were included in this study because the researchers had prior knowledge that they also administered medication in long-term care facilities (LTCFs) in the Western Cape province (WCP) (RSA 2005). This article is part of a larger study that focused on factors associated with the processes of safe medication administration in LTCFs, including nurses and organisational resources. Furthermore, to the best of our knowledge, no studies have investigated the link between medication administration processes and medication errors in the elderly in South Africa.

## Methods

### Study design

A non-experimental cross-sectional descriptive design with a quantitative approach was applied in this study. By using a self-administered questionnaire, data were collected simultaneously from three categories of nurse respondents with different levels of education in both funded and private LTCFs.

### Setting

This study was conducted in a natural, uncontrolled environment in 28 funded and private LTCFs in the WCP, South Africa. Funded facilities are state-subsidised, while private facilities receive no government funding.

### Population and sampling

The initial target population consisted of all 430 nurses (*N* = 430) working in all the LTCFs (*N* = 56) in the Metro-North, WCP, who were registered at the Department of Social Development (Department of Social Development 2015) as a provider of frail care in terms of the *Older Persons Act* 13 of 2006 (Republic of South Africa 2006). Stratified sampling was applied by dividing the *N* = 56 LTCFs into two strata of *n* = 20 funded and *n* = 36 private LTCFs, to include equal samples from both types of facilities. Each stratum was then randomised, and a 50% sample size of each stratum was selected, thus 10 funded and 18 private facilities. No further sampling was applied to include all the nurses in the randomly selected facilities.

### Data collection

A self-administered validated questionnaire was used with permission from Professor Ala Szczepura of the Warwick Medical School at the University of Warwick in the United Kingdom (Szczepura, Wild & Nelson 2011). The questionnaire was pre-tested on 17 nurses who met the inclusion criteria but excluding their results from the main study. Pre-testing was carried out to correct errors and modify the questionnaire to enhance its reliability, validity, and appropriateness for the South African context. The questionnaires included the following: introductory session, invitation to partake, a risk-benefit analysis, and the time frame needed for completion. It comprised seven sections: socio-demographics; policies, training, and medication administration; alterations to the medication administration record (MAR); and special circumstances in medication administration. The use of computers and mobile phones and sources of job pressures data were collected but not included in this article. Questions were close-ended and in Likert scale or dichotomous format. An online application from Google was used to create the online questionnaire. All data collection occurred between 12 June and 30 August 2020.

### Data analysis

The raw data were abstracted from the respondents’ responses to the questions in the questionnaire, then grouped and captured on a Microsoft Excel spreadsheet. Descriptive statistical analysis was performed to summarise and describe the collected data using the Statistical Package for the Social Sciences version 27 (SPSS 27) and with the assistance of a biostatistician from Stellenbosch University. The statistical tests included the Pearson Chi-square, the Pearson product-moment correlation coefficient, Spearman’s Rho 2-tailed statistical test, means, standard deviation, and Cronbach’s Alpha. The results are presented in tables, figures, frequencies, and bar graphs.

### Ethical considerations

This study was granted ethical approval by the Health Research Ethics Committee of Stellenbosch University (S19/10/252). There were travel restrictions following the declaration of a national disaster because of the COVID-19 pandemic and the subsequent imposition of a national lockdown (Department of Co-operative Governance 2020; Department of Co-operative Governance and Traditional Affairs 2020; RSA 2002). Consequently, the researchers applied for a minor amendment to the original proposal to include the online distribution of questionnaires in addition to the distribution of paper-based questionnaires. The questionnaires were pre-coded with a number for the facility, but respondents were not identifiable. Written permissions from the LTCFs were provided by the facility managers and informed consent by the respondents. The consent forms were available in English, Afrikaans and isiXhosa. All respondents chose to complete the consent form in English. Therefore, the questionnaire was only available in English, the accepted business language in the LTCFs in the WCP.

### Results

A total of *N* = 123 respondents took part in the study. This sample comprised RNs *n* = 60 (48.7%), ENs *n* = 35 (28.5%) and ENAs *n* = 28 (22.8%). These respondents self-reported on the availability of medication policies, the medication management processes they followed, the impact these processes had on the residents’ health status, and the implications of medication errors for the LTCFs.

#### Medication policies

About 85% of respondents reported that they had a recognised medication policy in their LTCFs to guide them. However, 27.6% indicated no specific periods specified for them to read these policies. Indeed, 13.8% reported that they were only required to read these policies upon employment at the LTCFs.

#### Medication management process

Of the RNs, 96% *always* knew the purpose of the drugs that they administered, while all of the ENAs, 100% reported that they only *sometimes* knew the purpose of the drugs that they administered. Relative to the size of the groups, more ENAs, 82.1%, conducted drug rounds alone than either the RNs, 76.7% or ENs, 60.0%. Of the RNs, 58.3% reported that they felt extremely at ease when conducting drug rounds out alone, while ENs, 42.9%, and ENAs, 75.0%, reported feeling fairly at ease. There was no significant linear correlation between comfort levels and years of work experience (*r* = 0.027, *p* = 0.765).

Regarding checks performed before medication administration, 95.9% of respondents indicated that they always performed glucose monitoring before administering insulin, 61.0% monitored blood pressure before administering antihypertensive medication, and 39.8% recorded the pulse rate before issuing Digoxin in their LTCFs. A statistically significant difference was found (*p* ≤ 0.001) when using the Spearman’s Rho 2-Tailed statistical test (correlation coefficient 0.409) between performing pulse checks and whether respondents received training in performing these checks.

About 31% of respondents did not check the contents of medication containers before administration because they assumed that the contents would be correct (Table 1). However, 82.9% of respondents indicated that they had encountered incorrect contents. In addition, 75.6% witnessed out-of-date containers, and (69) 56.1% saw medication from blisters/containers that were not added or removed when doses were changed. About 46% of the respondents witnessed residents missing medicines because they were absent during medication rounds, although only 14.6% of nurses made a note to indicate this on the MAR. About 83% of the respondents shared medication between residents mainly when the stock ran out (Table 1). The Pearson Chi-square test showed a statistical difference (*p* = 0.002) between the three nurse categories and how often they witnessed the practice of sharing residents’ medication. Relative to the size of the groups, it appeared that 24 (85.7%) of the ENAs in particular, saw this practice reasonably frequently.

**TABLE 1:** Medication management process.

Variables	RNs *N* = 60		ENs *N* = 35		ENAs *N* = 28		Total *N* = 123 (100%)	
	*n*	%	*n*	%	*n*	%	*n*	%
Performed glucose monitoring before administering insulin	56	93.3	34	97.1	28	100	118	95.9
Monitored blood pressures before administering antihypertensive medication	48	80.0	22	62.9	5	17.9	75	61.0
Monitored pulse rate before administering Digoxin	29	48.3	17	48.6	3	10.7	49	39.8
Assumed medication containers were correct so it did not warrant checking before administration	14	23.3	10	28.6	15	53.6	39	31.7
Witnessed incorrect content of medication containers	48	80.0	27	77.1	27	96.4	102	82.9
Witnessed out-of-date medication containers	43	71.7	24	68.6	26	92.9	93	75.6
Witnessed incorrect medication administration due to medication not added or removed when dose changes occurred	30	50.0	17	48.6	22	78.6	69	56.1
Witnessed medication missed due to residents absent during rounds	19	31.7	16	45.7	22	78.6	57	46.3
Witnessed sharing of medication between residents when their own stock ran out	50	83.3	28	80.0	25	89.3	103	83.7
Discussed medication after days off with colleagues rather than check MARs	49	81.7	27	77.1	28	100.0	104	84.6
Agreed that signatures are required for alterations to MARs	58	96.7	29	82.9	21	75.0	108	87.8
Witnessed alterations to MARs not signed by two people	50	83.3	24	68.6	26	92.9	100	81.3
Finding it difficult to decipher handwritings	48	80.0	28	80.0	27	96.4	103	83.7
Observed mass signing of MAR charts	33	55.0	16	45.7	20	71.4	69	56.1

RNs, registered nurses; ENs, enrolled nurses; ENAs, auxiliary nurses/assistants; MAR, medication administration record; %, percentage.

The results showed that 84% of the respondents prefer to discuss medication changes after their days off with colleagues rather than studying the MAR charts (Table 1). On the question of whether signatures were required when making alterations to MARs, 87.8% of respondents indicated that this was indeed so. The Pearson Chi-square test identified a statistical difference (*p* = 0.009) between the last training the respondents received and whether they thought a signature was required with alterations to MARs.

Regarding the lack of recording indicated with missing entries, 99 respondents (80.5%) believed the reason was that *people forget.* Similarly, 77% of respondents cited this same reason for non-administration, and 88% of respondents provided this reason for not recording the number/dosage of ‘pro re nata’ (PRN) medications. About 69% of respondents observed medication changes made on the same line in the MAR charts rather than new entries made. Furthermore, 81.3% of the respondents reported that these were not signed by two people. Most respondents, 103 (83.7%), appeared to find handwriting difficult to decipher. The mass signing of MAR charts (all charts signed together simultaneously) was observed by 56.1% of the respondents (Table 1).

#### Changes to residents’ health status

The respondents saw many medication errors that could have an impact on the health of residents. Of these, the medication that was omitted altogether was seen most often by 64.8% of respondents (Table 2). Following this error was the medication given at the wrong time, seen by 62 of the respondents (50.8%), and administering medication after it was discontinued, seen by 34 of the respondents (27.9%).

**TABLE 2:** Medication errors seen by respondents in their long-term care facilities.

Medication errors seen (1–6)	RNs, *N* = 60 (f)		ENs, *N* = 35 (f)		ENAs, *N* = 28 (f)		Total of all three nurse categories per variable *N* = 123 (100%)	
	*n*	%	*n*	%	*n*	%	*n*	%
Medication missed altogether	35	59.3	23	65.7	21	75.0	79	64.8
Medication given at the wrong time	24	40.7	19	54.3	19	67.9	62	50.8
Administering medications that have been discontinued	13	22.0	10	28.6	11	39.3	34	27.9
Wrong dosage being given	11	18.6	10	28.6	1	3–6	22	18.0
Medication given to the wrong resident	8	13.6	6	17.1	5	17.9	19	15.6
Wrong medication given	4	6.8	9	25.7	3	10.7	16	13.1

**Total of all six variables per nurse category (Total =** ***N*)**	**95**	**-**	**77**	**-**	**60**	**-**	***N* = 232**	

RNs, registered nurses; ENs, enrolled nurses; ENAs, auxiliary nurses; auxiliary nurses/assistants; MAR, medication administration record; f, frequency; %, percentage.

#### Implications of medication errors for long-term care facilities

About 96% of the respondents were fair to very confident that their residents received their medication on time, that the medication rounds were time-efficient, and that their systems were the best given the number of staff available to administer medication.

About 57% of the respondents cited the most common error of medication accountability as the failure to sign for medications that they indeed administered (Table 3). Relative to the size of the groups, this was the main concern for the ENs, 62.9%. The second most common error of medication accountability with 69 of the respondents (56.1%) was nurses not providing reasons for not administering medications. Relative to the size of the groups, this error appeared to be the biggest concern for both RNs, 33 (55.0%) and ENAs, 19 (67.9%), as shown in Table 3.

**TABLE 3:** Most common errors of medication accountability.

Common medication accountability errors (1–6)	RNs, *N* = 60 (f)		ENs, *N* = 35 (f)		ENAs, *N* = 28 (f)		Total of all three nurse categories per variable *N* = 123 (100%)	
	*n*	%	*n*	%	*n*	%	*n*	%
Not signing for medication administered	30	50.0	22	62.9	18	64.3	70	56.9
Not recording reasons for non-administration	33	55.0	17	48.6	19	67.9	69	56.1
Not recording actual amounts	8	13.3	13	37.1	13	46.4	34	27.6
Not recording times for PRN medications	26	43.3	16	45.7	16	57.1	58	47.2
Not booking in stock received	7	11.7	5	14.3	2	7.1	14	11.4
No witness available to sign MAR changes	21	35.0	14	40.0	8	28.6	43	35.0

**Total of all six variables per nurse category (Total =** ***N*)**	**125**	**-**	**87**	**-**	**76**	**-**	***N* = 288**	

RNs, registered nurses; ENs, enrolled nurses; ENAs, auxiliary nurses/assistants; PRN, pro re nata; MAR, medication administration record; f, frequency; %, percentage.

**FIGURE 1:**
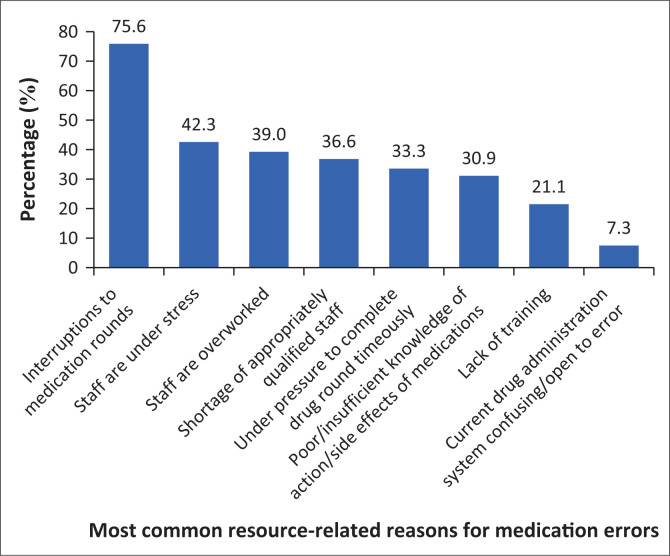
Most common resource-related reasons for medication errors.

The respondents were provided with eight close-ended reasons for resource-related medication errors and could choose more than one reason. As indicated in Figure 1, the reasons were reported as follows: frequent interruptions during medication rounds, as reported by 93 respondents (75.6%), staff being under stress, 52 respondents (42.3%), staff being overworked, 48 respondents (39.0%), a shortage of appropriately qualified staff, 45 respondents (36.6%), nurses who felt under pressure to complete drug rounds on time, 41 respondents (33.3%), poor or insufficient knowledge of the action of medications and their side effects, 38 respondents (30.9%), and a lack of training, 26 respondents (21.1%). Only nine of the respondents (7.3%) reported that their drug administration system might be confusing or open to error.

## Discussion

This research aimed to examine the medication administration processes followed by nurses as self-reported by them to identify possible medication errors. The conceptual framework used was Donabedian’s Structure-Process-Outcome Quality of Care Model (Donabedian 2005). In South Africa, the National Department of Health provides policy guidelines for medication management in LTCFs (Department of Health 2011). All medication must be prescribed by a medical practitioner or an authorised prescriber who must review the residents and the medication they receive six-monthly (Department of Health 2011). From the researchers’ experience, LTCFs do not employ medical practitioners. External medical practitioners will visit LTCFs if an agreement was negotiated, but residents are mostly transported to the medical practitioners at their private practices or to the clinics. Medication is obtained through various sources such as a medical practitioner, clinics, medicine depots, or pharmacies. Medications are dispensed in their original containers, blister unit-dose packs, or patient-ready medication packs preprepared at the hospital for state-subsidised residents. Long-term care facilities are allowed to keep small quantities of over-the-counter medication for minor ailments if the management thereof is addressed in a protocol signed by a medical practitioner (Department of Health 2011).

Some LTCFs used ENAs to administer medication, even though there is no provision for ENAs to administer medication in terms of Chapter 6 of Regulation 2598 of 1984: the Regulations Relating to the Scope of Practice of Persons who are Registered or Enrolled under the *Nursing Act*, 1978 as amended (SANC 1984). The scope of practice for nurses in South Africa is currently being reviewed, although not yet finalised (SANC 2021). Certain LTCFs had no medication policies, whereas some LTCFs with policies did not specify timeframes for reading these policies. The purpose of medication policies is to guide nurses and should be readily available and regularly consulted (Lindblad, Flink & Ekstedt 2017; Vogelsmeier 2011). Furthermore, faulty policies could contribute to 6% of medication errors (Ferrah et al. 2017). The Code of Ethics for Nursing Practitioners in South Africa also confirms that nurses are accountable for their actions and omissions (SANC 2013). Although the ENAs in this study felt fairly at ease when performing drug rounds alone, they also showed the most concern for the shortage of appropriately qualified staff as a reason for medication errors. A study by Aiken et al. (2017) showed that when one RN is substituted with one ENA per 25 residents, it reduced the skill mix from 66.7% to 50%, which could lead to a 21% increase in the probability of mortality for residents. The absence of medication policies for guidance and responsibilities beyond the scope of practice of specific nurse categories could leave nurses exposed. Another concern is the exposure of the LTCFs to vicarious liability. In other words, the employer could also be held liable for the actions of their employees in terms of common law (Dhai & McQuoid-Mason 2011).

The respondents’ knowledge of and training on medication was insufficient. All the ENAs participating in the study reported that they only *sometimes* knew the purpose of the drugs they administered. The respondents were also concerned about the insufficient knowledge of medications’ side effects and actions. This finding was in keeping with widely-documented evidence of nurses’ lack of knowledge of medication (Al-Jumaili & Doucette 2017; Dilles et al. 2011; Ellis et al. 2012; Szczepura et al. 2011). Tshiamo et al. (2015) attribute a lack of medication knowledge to the curricula that focus primarily on the theory in pharmacology courses, but do not provide students with sufficient exposure to practical, real-life situations. Despite mandatory medication training every six months (Department of Health 2011), only 19% of respondents received training on medication management over the last year.

Even though medication management includes at least five phases, the study of Odberg et al. (2019) on the nurses’ role in medication administration showed that nursing assistants viewed medication administration as being straightforward and comprising only two phases: preparation and administration. When focussing on the administration phase, there are ‘five rights’ to adhere to. These include performing checks to confirm that the right medication and dose is given to the right patient via the correct route at the proper time (Grissinger 2010). Transcribing is done by the nurses by writing down the prescribed medication from the original doctor’s prescription on the MAR. After administering the medication and monitoring the resident, the nurses document the process on the MAR (Ferrah et al. 2017). This study found that most respondents relied on colleagues for information about medication changes upon returning to work after days off instead of verifying changes on the MARs. The respondents also alluded to administering medications without checking the medication beforehand, based on the assumption that it would be correct, but often leading to administering the wrong medication, as shown in Table 1. Mistakes could be fatal in 9% – 15% of all instances when administering the wrong medication and administering medication to the wrong resident, especially if the residents were between 70 and 90 years old (Ferrah et al. 2017).

The medication error reported most frequently by respondents was that of medication omitted altogether; this was followed by medication administered at the wrong time. Medication errors such as the late dispensing of medication by pharmacists can often lead to secondary medication errors, such as the late administration of medication, which could change the residents’ health status (Carayon et al. 2014). Even though redundant medication must be removed and stored in separate secured locations until disposed of (Department of Health 2011), more than half of the respondents saw outdated containers where medication was not added or removed when the prescriptions changed. About 27% of the respondents alluded to administering medication after it was discontinued. Administering wrong dosages is especially harmful when administering antipsychotics, sedatives, anticoagulants, and antidiabetic medicines (Desai et al. 2013; Ferrah et al. 2017). Prechecks are also advised before administering certain medications, such as a pulse check before administering cardiac glycosides such as Digoxin, blood pressure checks before administering antihypertensive drugs, and checking blood glucose levels before administering insulin. When abnormal vital signs are found, the nurse should consult with a doctor before administering these medications (WHO & Regional Office for the Western Pacific 1998). Failure to perform prechecks adequately or appropriately, as in this study, could lead to administering wrong dosages with serious consequences. Al–Jumaili and Doucette (2017) illustrate the effect of an overdose of hydralazine, an antihypertensive drug, stating it could cause a lupus-like syndrome.

Interruption during medication rounds has been extensively documented by researchers as a potential cause of medication errors (Al-Jumaili & Doucette 2017; Dilles et al. 2011; Ellis et al. 2012; Ferrah et al. 2017; Metsälä & Vaherkoski 2014; Odberg et al. 2019; Qian et al. 2015). Interruptions could be described as active, such as answering phones or having discussions with colleagues and residents, or passive, such as bells ringing and background noises (Odberg, Hansen & Wangensteen 2018). However, nurses could become desensitised by these interruptions to the extent of accepting them as normal occurrences (Odberg et al. 2019). In this study, the respondents also experienced pressure to complete drug rounds within a certain amount of time. According to Ellis et al. (2012), this pressure can lead nurses to take shortcuts. Those authors found that nurses admitted to taking shortcuts by disguising medicine in fruit juice to facilitate swallowing. This enabled the nurses to complete a drug round on time without the risk of action against them by supervisors. Preston et al. (2014), however, states that the metabolism of medicines may be affected by certain fruit juices.

The respondents witnessed various record-keeping errors, such as the following: MARs being changed and signed by only one person rather than making new entries; not signing at all for medication administered; not recording reasons for non-administration; not recording times for ‘pro re nata’ medications and the mass signing of charts (all charts signed together at the same time). The main reason for poor record-keeping, according to the respondents, was that ‘people forget’. Andersson et al. (2018) also found incomplete or lacking records as the most common factors contributing to medication errors. These writers’ results were consistent with the statements by the World Health Organization to the effect that inaccurate MARs are organisational factors in care homes that contribute to medication errors (WHO 2016). Furthermore, illegible handwriting is a factor that could lead to medication errors. Metsälä and Vaherkoski (2014) stated that unclear handwriting is associated with prescription factors which could cause an administrator to be unable to read the orders correctly, leading to potential errors.

## Limitations

The setting of this study may differ from that of developed countries in terms of the characteristics such as nursing homes, acute care facilities, and skilled care facilities. Also, only one of South Africa’s nine provinces was included in the survey, which may reduce the generalisability of the results. The unforeseen COVID-19 pandemic had a negative effect on the data collection process and led to a relatively small sample size. There were also insufficient formal reporting systems in the LTCFs. Therefore, the researchers excluded formal incident reports from the study. When comparing responses by the category of nurses, there is a possibility that the RNs may be more cautious about describing practices or their concerns which may lead to social desirability bias.

## Conclusion

Nurses self-reported the medication administration processes they followed in the LTCFs. The factors associated with medication errors in the elderly included the following: the sharing of medication between residents; omission of doses; administering medication at the wrong times; failure to conduct pre-checks before administering medication; and neglecting to sign after medication administration. The nurses reported the following challenges: being frequently interrupted during medication rounds; feeling under pressure to complete drug rounds on time; being under stress and overwork; experiencing a shortage of appropriately qualified staff; and having poor or insufficient knowledge of the action of medications and their side effects. The researchers recommend that LTCFs develop risk management strategies aligned with the scope of practice of the specific categories of nurses. Monitoring adherence to mandatory training and the procedures followed are needed, with the inclusion of medication errors as topic in quality management programmes.

Further research is recommended to examine the workflow processes for medication administration in the LTCFs. Also, the development and implementation of formal reporting systems for medication errors can provide valuable insights into the prevalence of medication errors to assist in developing risk management strategies and policies in the LTCFs. On a national level, forming patient safety organisations is recommended to analyse medication error reports, perform root cause analyses, and implement preventative strategies. South Africa could also benefit from the membership of international organisations which strive to improve medical practices. This research study confirmed the most important categories of medication administration errors and their causes, as perceived by nurses working in LTCFs in the Western Cape. Therefore, it fills a gap in the knowledge of risk factors associated with medication administration processes followed by the nurses in LTCFs in the South African context using self-reported questionnaires.
